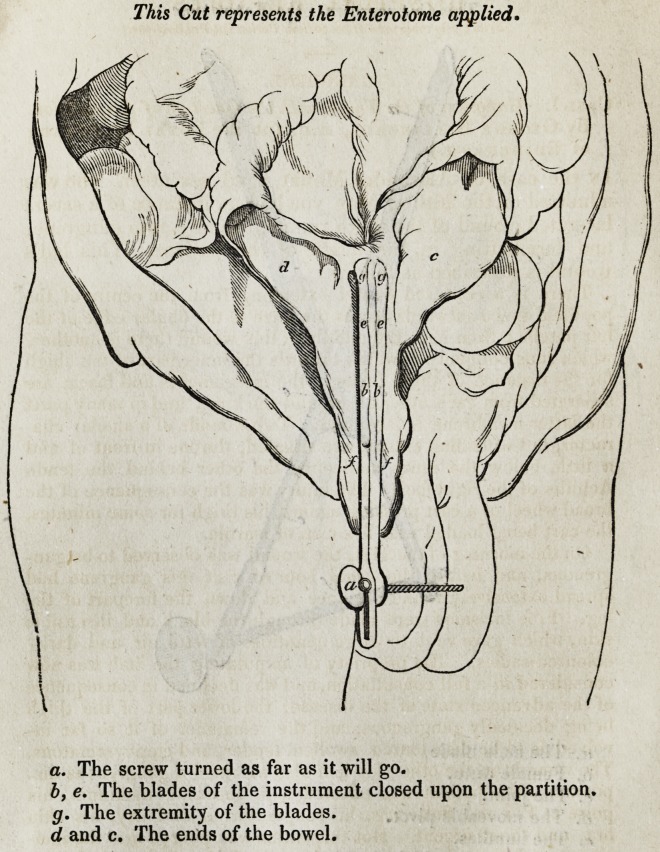# Memoir on a New Method of Curing Artificial Anus

**Published:** 1828-09

**Authors:** M. Dupuytren


### ARTIFICIAL ANUS.

*Memoir on a new Method of curing Artificial Anus.* By M. Dupuytren. Condensed from the *Memoires de l'Academie Royale de Medecine;* and illustrated by Wood-cuts.

Artificial anus has been generally looked upon as incurable; but I trust that, from the details I am about to give, it may hereafter be ranked among those maladies which admit of relief, without much difficulty or danger.

It was necessary that I should first ascertain the exact anatomical condition of the parts in this affection. In the natural state, the aliments traverse, in a given time, the whole length of the intestinal canal, and undergo, in each of its parts, a series of changes; as the result of which, they furnish the elements of nutrition; after this, the residue passes on, and is expelled by actions which are under the control of volition.

The successive elaborations, the absorption of the chyle, and the evacuation of the residuum, constitute a series of conditions indispensable to the regular action of the alimentary canal. Hence it happens that if, in consequence of any disease, these numerous conditions are altered, the digestion becomes disturbed, and more or less diminution of nutrition results. This is what takes place in preternatural anus; a malady which consists either in an original or accidental aperture in the alimentary canal, at a point different from the proper anus, by which the aliments, or feculent matters, are evacuated involuntarily, and before they have undergone the necessary changes. The opening is rarely congenital, but almost always results from injuries, with or without loss of substance, inflammations, abscesses, and hernia, terminating in the destruction of a portion of the intestine. I mean only to treat of the latter variety, artificial anus.

This condition is by no means easily produced; and, even where life can be preserved only by this means, nature and art united often fail to overcome the difficulties opposed to it.

The establishment of an artificial anus requires the co-operation of many circumstances. It is necessary that the intestine in which the new anus is to be formed, be placed opposite to that part of the abdominal parietes through which the matters are to make their exit; that it admit of being kept in this situation, or, still better, that it be fixed in the opening; that a ready communication can be kept up between this external aperture and that in the bowels; and, above all, that these be capable of forming adhesions to the neighbouring parts: circumstances, the simultaneous occurrence of which are rare. Once established, the artificial anus presents an opening formed at the expense of the intestine and abdominal parietes, intimately united together. This opening, generally round, but occasionally irregular, varies in size, from a few lines to an inch or more in diameter, and is surrounded by radiating folds of the skin plaited upon itself. The border presents a cicatrix, uniting the skin of the belly to the mucous membrane of the bowel.

These adhesions are the result of inflammation, and always commence in the serous surfaces of the intestine and abdominal cavity; and thence extend to the other textures, soon reaching the skin and the mucous membrane. In hernia, the adhesions precede the destruction of the parts, and thus prevent the escape of the alimentary matters into the abdomen. In wounds, again, they do not take place till after the division of the intestine; and this is the reason why these are so frequently fatal. Their extent varies: it is from half a line to a line, in most cases; but in others it is several lines, and sometimes, though rarely, half an inch. But, as these adhesions never extend very far along the intestines, it results that a cul-de-sac is formed, the opening of which presents towards the belly, and the bottom of which corresponds to the skin. Into this cavity the viscera are protruded, in some individuals, so as to produce herniæ, obstructing, or even altering, the position of the artificial anus.

The opening of the anus is almost always occupied by some part of the internal membrane of the bowel, irregularly puckered. Not unfrequently protrusions of the bowel take place, the mucous membrane becoming irritated and inflamed. This generally occurs at the upper end of the intestine, sometimes at the lower, and occasionally at both at once; but the eversion always forms a curve, owing to the shape and resistance of the mesentery. Its length varies from one to fifteen or more inches; and it may be easily imagined how much it must add to the pain and inconvenience.

Between the opening of the skin and the bottom of the artificial anus, there is a kind of funnel-shaped cavity, which SCARPA has described. This is formed by the various parts having been brought to a state identical with that of mucous membrane. The skin forms its border, the intestine its base. Its length, direction, form, and dimensions, vary infinitely, and have very great influence on the cure of the artificial anus. The greater the capacity of this funnel, the greater in general the disposition on the part of nature to cure the infirmity, or to second the efforts of art in her doing so.

It is in the bottom of this cavity that the most remarkable and important circumstances exist. There the orifices of the two extremities of the intestine, and the partition which separates them, are to be found. Of these openings, one belongs to the part of the intestine; and, in consequence of the intestinal matters always passing through it, it is the larger and freer of the two. The other orifice belongs to the inferior extremity of the intestine; and as it does not receive any, or at all events but very little, of the above matters, it is generally narrow and puckered.

Beyond these two orifices are the two extremities of the intestine, of which they are the terminations. These extremities, which are villous, and covered with mucus internally, and with serous secretions externally, retire into the abdomen, sometimes crossing and sometimes parallel, but most frequently separating from each other at a greater or less angle.

Between the two orifices is a projecting angle, more or less marked. This projection is produced by the union of the sides of the intestine. Formed by the part of the bowel which has been spared on the side next the mesentery, this projection juts forwards, nearer to or farther from the skin, according as the intestine has suffered a greater or less loss of substance, and undergone more or less change in its situation. It is small when the intestine has only just been pierced by a wound or slough, and when it runs along the posterior surface of the parietes of the abdomen in the natural direction of its curve. But it is very great when the whole circumference of the intestine has been destroyed, and when, in consequence of this, the two extremities meet at a sharp angle, and, *à fortiori*, when they are parallel. In the former case, there exists between the two orifices a kind of gutter, which may direct the matters from the upper one towards the lower: this, therefore, is the kind of preternatural anus most easily cured. In the latter case, there is no vestige of this gutter; and the projection of which we speak, placed between the two ends of the intestine, forms a barrier which the intestinal matter can neither break down nor pass: this is the kind of preternatural anus most difficult to cure.

This projection does not divide the funnel into two equal parts; or, if this be the case at first, it does not long continue. Thrown aside by the passing current of matters from the upper portion of the bowel, it becomes applied to the lower orifice, acting as a valve: hence the difficulty often experienced in finding the lower opening.

This projection, examined from the cavity of the intestine, has the form of a crescent, the angles of which presenting from the concavity towards the convexity of the bowel, are lost on the inside of the gut, or on the borders of the artificial anus. Examined from within, it is seen to unfold itself, and the two parts of which it is composed receive the mesentery between them. This division of the buttress at its base is the result of its mechanism: it is not formed of one single wall, except at its sharp edge; at every other part it consists of two sides, which separate from each other on entering the abdomen.

From this it results that the openings of the two ends are separated by a double partition; that, in order to pass from one of these openings to the other through the intervening partition, it is necessary to traverse the peritoneal cavity. Hence arises the difficulty and danger of attempting to establish a communication between the two portions of the canal through the projection which separates them.

The buttress and double partition are not fixed so firmly but that they can advance or recede: they are attached to the mesentery, and follow to a certain extent the movements communicated to them by it. The distribution of the mesentery merits consideration. Stretching from the anterior part of the vertebral column to the concave part of the intestines, it has, in the natural state, no greater extent than between those two points: it is always more or less dragged when the intestine leaves its natural situation, as in most cases of hernia and penetrating wounds of the abdomen, with protrusion of the bowels. Compelled to follow the gut which is displaced, it forms a kind of cord from the vertebral column to that part of the bowel most distant from it. This cord is tense, and bends the body forward. This is particularly observed in cases of hernia which are adherent. In consequence of this distribution of the parts, the projection or buttress which has been described, as well as the intestine itself, is constantly pulled inwards by the mesentery; and hence we easily perceive the influence which the position and movements of the body must have on the cure of this malady. This dragging, however, is not free from danger, as I have known it sufficient, in two cases, to destroy the adhesions which united the extremities of the bowel to the parietes of the abdomen, thus producing effusion into the peritoneum.

Several individuals, cured of artificial anus without operation, having entered the Hôtel Dieu after several years, and having died of diseases unconnected with this, I examined the parts, and, in place of finding the intestine fixed to the inner surface of the abdominal parietes, I found it free and unattached! I might have suspected some mistake, had I not found a fibrous cord extending from the intestine to the part of the abdominal parietes corresponding to the artificial anus. Thus the efforts of nature were not limited to closing up the preternatural opening: they had even separated the intestine from the parietes of the belly; they had restored its natural curve and mobility, by elongating the cellular substance in the form of a cord.

Nor are these the only changes which take place. The upper extremity of the bowel, excited by the passage of the intestinal contents, acquires increased activity and size; a change in which the mesentery and lymphatic glands participate. The lower portion, on the other hand, ceasing to perform its functions, gradually wastes, till at length one part of the canal resembles that of an adult, and the other that of a new-born infant. Nevertheless, the lower end does not become obliterated, nor is it even entirely empty: it is filled to a certain extent with the usual intestinal secretions, which are converted into a white mass, of a soft consistence and albuminous appearance, and which may remain, without undergoing decomposition, for months or years, till it is either voided by a natural effort or washed out with enemata.

In the natural state, the intestine free, and floating in the abdomen, though attached to the mesentery, describes a series of curves, along which the contents pass without difficulty; but no sooner is an artificial anus established, than a portion of intestine, directed towards a particular point of the abdominal parietes, forms a triangle, the base of which is towards the mesentery, and the sides of which are formed by the upper and lower extremities of the bowel.

With regard to the evacuation of the intestinal contents by the artificial anus, the opening is not surrounded by any muscular apparatus capable of acting upon it at will; and the aperture is, therefore, always open to the matters which are constantly arriving. Besides, even if there were the necessary muscular arrangement, the contents of the bowels, deprived of a reservoir where they can be retained and formed, would constantly require to be voided. There is thus a constant flow of matters, varying according to the state of digestion and the situation of the opening; and hence the person of the patient is affected with an offensive smell, and the parts are liable to excoriations, &c. All the contrivances to obviate these evils, do so very imperfectly; and compression, so as to retain the matters within the bowels, often gives rise to such mischief as to render its abandonment necessary.

Almost all preternatural anuses which consist of simple perforations of a point in the circumference of the intestine, whether attended by hernia or not, are curable ; and we also succeed very frequently in those cases of artificial anus in which a third, or even half, the circumference of the gut has been destroyed for a few lines, or even an inch ; but, when the loss of substance embraces more than two-thirds or three-fourths of the circumference, the cure becomes proportionally difficult; for then, from the contraction in the gut, the buttress and partition become very prominent and formidable obstacles to the passage of the fecal matters. The result of the cases that have occurred to me, as well as of those which I have collected from different authors, is that the artificial anuses susceptible of cure are to those which obstinately resist every method as three to one : that is, two-thirds are cured by the ordinary methods, and the remainder require a more efficacious plan of treatment. The difficulties that oppose themselves to the cure are the loss of substance, and consequent contraction of the gut; the adhesion of its extremities; the changes in its direction and mobility; but especially the projection and double partition placed between the two extremities.

The loss of substance cannot be repaired, and it is necessary to respect the adhesions, so that it only remains to attack the partition and buttress.

It would seem, at first sight, that the simple section of these parts, either by the scissors or other cutting instrument, would be sufficient to re-establish the communication between the two ends of the gut; and such would be the case if the two sides of the projection adhered together; but a moment's reflection will shew that this operation would produce almost immediate death, by effusion into the cavity of the abdomen.

The two ends of the intestine which form the artificial anus are covered on all sides by the peritoneum, and this membrane forms a cavity around them. This circumstance, which forms an insurmountable obstacle to an immediate division, affords the very means by which the double partition separating the intestines may be divided without opening into the cavity. One of the most remarkable properties of serous membranes is to form adhesions when inflamed, and kept in contact: if, then, an inflammation could be excited between the two surfaces of the intestines, I conceive that I should afterwards be able to perforate and divide the parietes of these intestines, and establish a communication between the two extremities, without the risk of effusion.

My first idea was to pierce the partition with a needle, which would convey a thread to fill up the void that had been made: this thread, after having excited inflammation, might afterwards be replaced by a skein, increasing in size from day to day; so that, after some time, it might be large enough to destroy the partition entirely. Their cavities would then become reunited, and means might be adopted, without inconvenience, to prevent the passage of the feces by the artificial anus. These suggestions were the result of observation only. I wished to strengthen them by direct experiment upon living animals: with this view, I traversed the intestinal canal of several dogs with needles armed with threads, which I left in the wounds, returning the intestines into the abdomen. No effusion took place in any instance; the wounds and threads, after some time, were found surrounded by adhesive inflammation; the ligatures were either voided by stool, or taken away by gently pulling them; the openings made by the needles, and those in the parietes of the intestine, were always found closed, adhesion having taken place between the peritoneal coat of the punctured intestine and the neighbouring parts. A still more decisive experiment, attended with the same result, was made by forming an artificial anus in a dog.

In May 1813, a man named Aucler was admitted into the Hôtel Dieu, thirty-six years of age, who had laboured under strangulated hernia for five days, the consequence of which was the formation of an artificial anus. At first pressure was tried, but this produced symptoms so severe as to compel me to abandon its use. An attentive examination shewed me that the two extremities of the gut were perfectly on a level, and that their orifices were only separated by a projecting buttress and partition. After considering the best method of perforating this, I determined to pass a needle through it, leaving in the thread with which the needle was armed. The operation was short, and not very painful. Some days afterwards a skein was carried, by means of the thread, into the opening made in the partition. The size of the skein was increased at each dressing; and eight days after pains were felt in the abdomen, and some feces passed by the anus. Encouraged by this, the size of the skein was increased, till it produced a laceration of the buttress: this caused no ill effect, but still stercoraceous matter continued to pass from the artificial anus. Considering that those parts of the partition situated above the opening made by the needle might adhere together, and might be divided with as little danger as the parts situated below, this part was divided, half a line at a time, with a pair of blunt-pointed scissors, directed upon the forefinger. This was done at intervals of three or four days; and the incisions, very cautiously made, and which never passed the limits of the adhesions, enlarged the communication so much that all the feces soon came away by the natural anus. Compression was then used upon the artificial anus, and would most probably have closed the opening, but the man, wishing to hasten the cure, urged me to make a fresh attempt, and I had the weakness to do so. Some irregular portions situated round the aperture were tied, and excised: I afterwards carried the division of the partition higher than had yet been done, and in a few hours the patient was seized with acute peritonitis, which proved fatal. I apprehended at first that this inflammation might have been produced by the effusion of fecal matter into the abdomen; but, at a public examination of the body, no such effusion was found. The cavity contained merely a quantity of purulent serosity and albuminous flocculi, the ordinary products of acute inflammation. The communication between the two extremities of the gut was re-established for the space of about two inches. The ends, before separated, had now but one wall and one cavity; along the whole length of which, both before and behind, there was a raphe, produced by the cicatrix in the partition; and every thing announced that, had not this unfortunate accident intervened, the artificial anus would have been cured.

Chagrined at the result of this case, I again reviewed the question; and I was confirmed in my opinion, that establishing a communication between the two ends of the intestine, by destroying the partition, was the only mode that promised any chance of success, and that the sole defect was in the means hitherto employed.

It became necessary to devise a method of keeping the parts in contact previously to dividing them, and which would not effect their division until adhesion had taken place. At length, after many trials, upon the dead body as well as upon living animals, I believe that I have discovered the instrument which I sought for. It is composed of three pieces,—two branches and a screw. Each branch is about six or seven inches in length, and one, which may be called the male, because it is received into the other, has a blade four inches long, three lines broad, and half a line thick at its edge, which is undulated and terminated by a spheroidal button. At the union of the blade with the handle is a mortise, some lines in extent; behind this mortise is a handle, one, two, or more inches long, having another mortise running nearly the whole of its length, about three or four lines broad. The female branch is not quite so long as the former: it is composed, at one of its extremities, of two blades of the same length, breadth, and thickness as the male blade: between these two blades is a sort of sheath, to receive the other blade. At one of the ends of this blade is a cavity, to receive the button of the other. At the junction of the blade with the handle there is a moving pivot, which is to be received into the mortise of the other branch; the handle is terminated by a hole to receive the screw.

The third part of the instrument is a screw of several threads, an inch and a half long, terminated by an oval plate. This screw is to be placed in the mortise of the male branch of the instrument, and fixed in the female branch: its use is to separate or close at pleasure the two blades of the instrument. This instrument I named an *Enterotome*.

The application is easily understood: two branches, which may be separated or united at pleasure, provided with blades, very blunt, and with a waving edge, are moved by means of a screw passing across the handle. Whatever these blades enclose, is retained by them by means of their form, as well as by the introduction of the one into the other. The pressure which they exercise upon the parts they embrace has the effect, at first, of placing them in contact, and it may afterwards be increased so as to destroy their vitality. This instrument has not since undergone any alteration, but has been applied to every case of artificial anus upon which it has been necessary to operate. However, before I employed it upon man, I applied it to other animals, and upon each occasion it succeeded in dividing the parts in six or eight days. In every case where serous membrane was confined within the branches of the instrument, the parts were united by the second or third day, and consequently long before the solution of continuity, which does not happen till the seventh or eighth day.

The action of the enterotome was never attended with severe pain, and the inflammation was always confined to the immediate vicinity of the parts laid hold of. It did not produce solution of continuity like a cutting instrument,—that is, without any loss of substance: on the contrary, it caused mortification of the parts embraced by it, and a slough, which, when it separates, is always between the blades of the instrument.

The following is the first case in which the enterotome was employed:

—— Menage, ætat. twenty-six, had suffered from his infancy from an inguinal hernia on the right side, which became strangulated on the 2d January, 1815. On the sixth day, after vain attempts at reduction, the operation was performed. The intestine was in a state of mortification, and the feces passed by the wound. An artificial anus became established.

At the end of a year he was admitted into the Hôtel Dieu. The artificial anus was about half an inch in diameter: it was surrounded by irregular tumors, arising from the puffing up of the mucous membrane of the intestine, behind which, whenever the patient made the least exertion, a hernia appeared, giving rise sometimes to the invagination of the intestine. The neighbouring skin was extremely irritated; the man suffered great pain, and the stench he emitted was excessive. My firse step, after appeasing the irritation of the skin, was to determine the position of the two ends of the intestine. At length I discovered the direction of the extremities, as well as of the buttress and partition; and immediately I introduced the blades of the enterotome, separately, to the greatest possible height, into each of these ends; and, after having fastened them together, I closed them moderately. The patient experienced no pain: they were tightened on the following day, and some colicky pains ensued. In a few days the blades of the enterotome became a little moveable. About the sixth day, there were abundant evacuations by stool, and the instrument fell off on the eighth: the blades contained nothing but a membranous band, in which all the tunics of the parietes of the gut were cognizable. The length of this membrane, which was as thin and dry as parchment, was twenty lines, by two in breadth: this was the exact measure of the depth to which the instrument had been conveyed, and consequently that of the loss of substance which the partition of the intestine had undergone. From this time all the feces passed by the natural anus, and their escape by the artificial one could be prevented by pressure. Various methods were tried, without avail, to heal this up. At length, seeing the obstinacy of this opening, now little more than a line in diameter, I excised the edges, and brought them together by the twisted suture; and afterwards employed pressure. At length, after four months' labour, I had the pleasure of presenting this patient to the Faculty of Medicine, entirely cured.

In order to apply the instrument, it is necessary, first, to seek for both the orifices of the intestine, and to determine exactly their direction. This is the most difficult part of the operation. The upper orifice is, indeed, easily found; but, to discover the lower, the finger or a probe must be employed often for several successive days. These points being ascertained, and the patient placed upon his back, one of the blades of the enterotome is directed, by means of the index finger, into one of the orifices of the gut, according to the nature of the case, one, two, or three inches in depth. This blade is then given to an assistant, and the second blade is introduced, with the same precautions, and to the same depth, into the other extremity of the intestine; the two blades are then brought together, and articulated in the manner of a pair of forceps, by putting the tenon of the one into the mortise of the other. It is sufficient, at first, to take hold of the intestine, and to bring the blades of the instrument together in the same way as with a pair of scissors. The action of the enterotome being intended to be slow and gradual, it can only be kept up by mechanical means. This is done by the screw; and the pressure ought to be so managed as to destroy the life of the part from the first day: it is by so doing that the pain and inflammation are prevented. This pressure is to be increased every other day, by giving the screw a turn or two. It might appear, at first sight, that an instrument carried to such a depth into the abdomen, and pressure made to such an extent as to destroy the parietes of the intestines, would produce colic, vomiting, inflammation, and other severe accidents; but such has not been the case. Indeed, those to whom the instrument has been applied have experienced but very slight pain: a very small number have suffered from colic and vomiting; the inflammation has been confined to the portion laid hold of by the instrument, and has not been communicated to other parts. After a few days the instrument becomes a little moveable: this mobility increases daily, until it falls off without any pain or bleeding; and this happens always between the seventh and tenth days. When it has fallen out, the blades are found nearly closed, containing within them a membrane similar to that above described. The most difficult part of the cure remains, that is, to obliterate the external opening; and many weeks are requisite to accomplish this.

The following case proves that the above plan is equally applicable to those instances of artificial anus resulting from wounds.

Louis Tubert, aged forty-two, was admitted into the Hôtel Dieu, March 1824, with an artificial anus. This man was of weak intellect and small stature, with a muddy complexion, extremely thin and feeble. Eighteen years before, he had produced a rupture at the ring of the left side, in consequence of a violent exertion. The size of the tumor increased, so that, at the end of fifteen years, it was as large as an infant's head, and was in a great measure irreducible.

Believing himself to be an object of ridicule on account of his infirmity, Tubert thought to rid himself of it by an operation. He made a large incision in his scrotum, opened the hernial sac, and exposed a knot of intestine eighteen inches long. He then became alarmed, and sent for a surgeon, who with some difficulty reduced the gut; but the hernia remained; for, considering a bandage as merely a palliative cure, he refused to wear one. He still continued to imagine that he could cure himself by an operation; and, brooding over this for about three years, at length, on the 22d February, 1824, he made another incision into the scrotum, opened the hernial sac, and, bolder than on the former occasion, he laid hold of the intestine, and cut off a piece of it. The pain, bleeding, and issue of fecal matter, however, alarmed him, and he once more sent for his surgeon, who enlarged the opening in the scrotum, discovered the two extremities of the divided gut, and reunited them by several points of suture. These failed in uniting the intestine, but they produced inflammation of its extremities, which united them to the lips of the wound, and thus an artificial anus was formed. The part removed was two inches and a half of the small intestine: it did not form a complete cylinder, but was interrupted at two parts, one for the extent of about half an inch at its extremity, and the other about the centre.

On his admission into the Hôtel Dieu, there was found, on the left side, a long tumor, extending from the ring to the bottom of the scrotum: it was hard, shining, partly reducible, and exhibited, at its lower and anterior part, a wound of a vivid red colour, formed below by the scrotum, and above by the two ends of the intestine twisted upon each other so as to make several turns. They were placed side by side: that on the right gave vent to some fluid feces, mixed with undigested matters: this was continual and involuntary. The other end of the intestine was retracted, and did not discharge any thing. The patient was in a filthy state, and suffered from colic, as well as from a fixed pain and tension in the left iliac region. After the lapse of a few days, the two reverted ends of the intestine were reduced, and a bandage applied over the artificial anus: enemata were then administered, and a regular diet established. Pressure could not be borne: it was tried several times, but always occasioned symptoms rendering it necessary to abandon its use.

The man continuing to waste, I determined to seek for the two extremities of the gut. I found that the upper, or stomach end, was situated at the bottom of the scrotum, where it formed inextricable circumvolutions, and that the rectal end led directly to the ring. This situation of the upper end of the gut caused me to hesitate as to any attempt at a radical cure; but at length the urgent entreaties of the patient, who had heard that similar infirmities had been cured, induced me to make the attempt, especially as I dreaded his making an attempt at a third operation himself.

I accordingly proceeded on the 31st May, in presence of MM. Larrey, Aumont, and Sanson, to introduce the blades of the instrument separately into each extremity of the gut, passing them in as deeply as possible. The upper blade could only be carried to the depth of from two and a half to three inches, and in this situation I was obliged to fix the instrument. On the first day there was no pain; the next day there was an œdematous swelling and some redness at the edge of the artificial anus, but still there was no pain. On the sixth and seventh days, slight colic was felt; the eighth day, the instrument fell off, and the two extremities of the intestine formed one canal. From this time clysters were administered every day; flatus passed per anum, but the feces still made their way by the artificial anus, and therefore the patient continued still to become thinner and weaker.

After the lapse of a fortnight, Tubert conceived that he had passed feces by the natural anus, and the volume of the tumor diminished. Some time after this, pains in the belly began to be felt: their violence at first threatened to exhaust the patient's remaining strength. However, the evacuations became established in the proper channel; they acquired regularity; and strength was in some measure restored. The size of the tumor gradually decreased, but still some fecal matter passed by the artificial anus. To arrest this entirely, I applied an apparatus for the purpose of holding the lips of the wound in contact. This compressor was composed of two segments of a circle, of equal size, a few inches long, and some lines only in breadth, placed parallel to each other, each surmounted by a shank of an inch and a half high: these shanks were united by a crosspiece fixed to one, and moveable upon the other, which received it in a groove with which it was pierced. Beneath this crosspiece was a screw, which rested upon one of the shanks and moved upon the other; and the movements of which to the right and left produced, as required, either the separation or approximation of the compressing arches. These being padded, were separated; the skin in the vicinity of the artificial anus was raised up, and the fold which it formed was insinuated between the arches; a slight motion given to the screw from right to left brought these segments of the circle together, and thus the artificial anus became so compressed that nothing could pass through it. When this compressor was applied to Tubert, it happened as I expected: nearly all the fecal matter took its natural course; the little that still continued to ooze out was suppressed by an increased adaptation of the instrument; and then, for the first time, a smile was seen upon the patient's countenance. The instrument, however, sometimes got loose, and at others produced excoriation of the parts, and then the feces began again to flow from the wound; and, as this occurred several times, gentle and constant pressure with a bandage was substituted.

From the period that the excrement passed by the natural channel, the patient rapidly recovered his flesh and strength, so that his appearance was sufficient to shew whether there had been any discharge from the artificial anus or not. A triangular flap of skin, situated at the upper part of the artificial anus, resulting from the irregular cut made by the patient, seemed well adapted to close what remained of the aperture: this flap, as well as the edges of the opening, was therefore touched with lunar caustic; and it was then applied and maintained in this position by the assistance of a bandage. The flap united, and completely closed the opening; thus perfecting the cure in rather less than five months.

I could multiply the examples of cure by the method above related, but the detail of a number of cases would add nothing to what I have already said: it will be more useful to give the general result of these operations. The result, then, of the facts collected in my own practice, as well as of those communicated to me, or published by different medical men, is that forty-one operations for artificial anus have been performed by means of the enterotome; viz. twenty-one by myself, and twenty by other practitioners, among whom is M. Lallemant, professor at Montpelier. Three-fourths of these operations were rendered necessary in consequence of gangrene from strangulated hernia; the other fourth in consequence of wounds, with loss of substance. Of these forty-one operations, three only have been fatal: one from a presumed effusion into the abdomen, one from indigestion, and one from peritonitis. Of the thirty-eight remaining patients, by far the greater number experienced no serious symptoms: some few were affected with nausea, vomiting, or pains in the belly, but these were remedied by simple means. The whole number were not equally well cured. Nine had, in spite of every thing that could be done, fistulous openings, which have obliged them to wear a bandage. Twenty-nine have been radically cured. Thus, the operation has caused death only in one case out of fourteen; and if the death by indigestion, which ought not reasonably to be attributed to the operation, be excluded, the proportion is reduced to one in twenty.

*This Cut explains the Anatomy of the Artificial Anus.*

*a.* Opening of the artificial anus, where the mucus membrane and skin meet.

*b.* The upper portion of the intestine.

*c.* The lower end of ditto.

*d.* The projection or buttress.

*e, e.* Coats of the intestine forming the double partition.

*f.* The ligament formed by the mesentery.

*g.* The infundibulum between the peritoneum covering the intestine and that lining the abdominal cavity; where herniæ are apt to form.

*This Cut represents the Enterotome.*

a. The male blade.
b. Female ditto.
c. The joint.
d. The moveable pivot.
e. The handles.
f. The screw by which the blades are made to approximate.

*This Cut represents the Enterotome applied.*

*a.* The screw turned as far as it will go.
*b, e.* The blades of the instrument closed upon the partition.
*g.* The extremity of the blades.
*d* and *c.* The ends of the bowel.

## Figures and Tables

**Figure f1:**
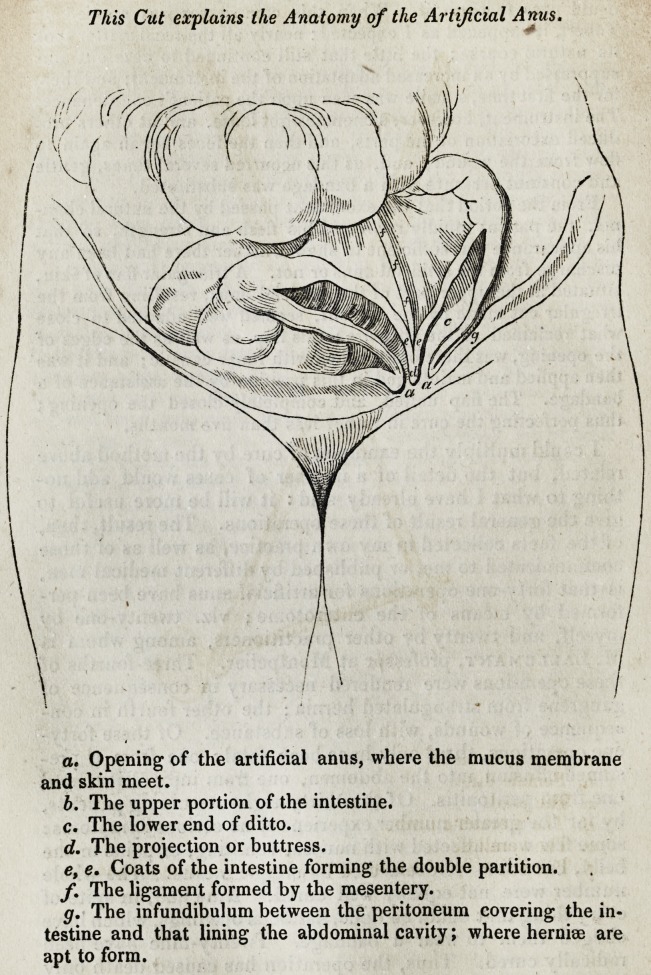


**Figure f2:**
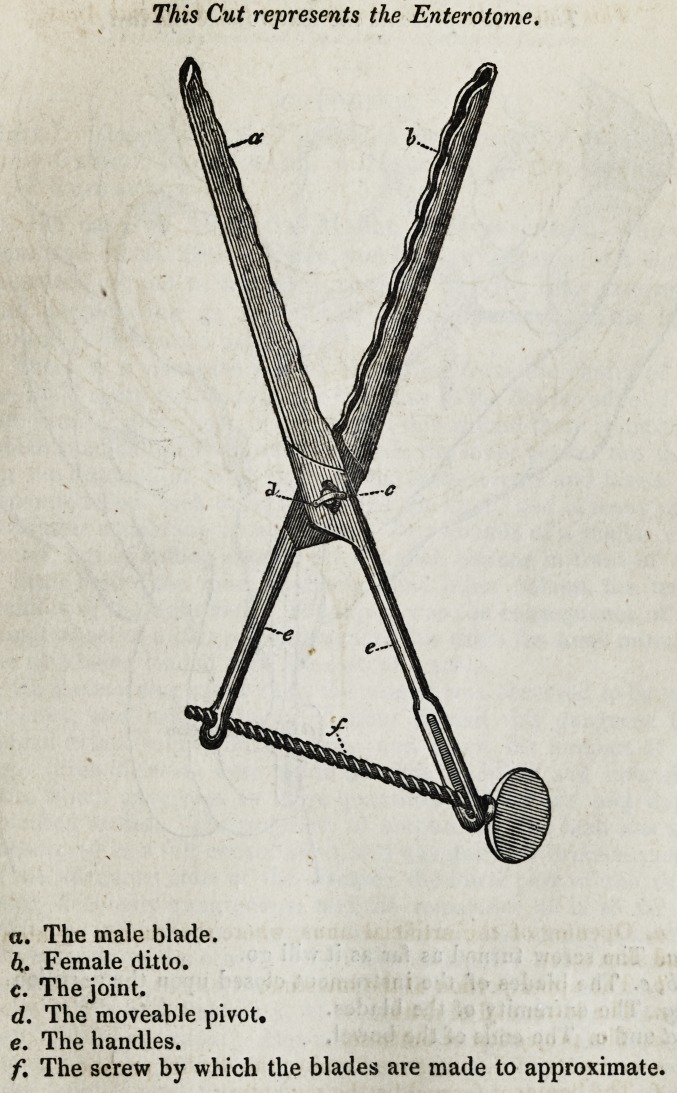


**Figure f3:**